# Genomic characterization of IncC plasmid-mediated triple carbapenemase-producing Providencia hangzhouensis ST222

**DOI:** 10.1099/mgen.0.001876

**Published:** 2026-11-10

**Authors:** Odion O. Ikhimiukor, Catharine Prussing, Kailee Cummings, Ashley L. Marcinkiewicz, Spencer Y. Kennedy, Jenna Cebelius, Elizabeth J. Nazarian, Kimberlee A. Musser

**Affiliations:** 1 Wadsworth Center, New York State Department of Health, Albany, NY, USA; 2 Vermont Department of Health, Waterbury, VT, USA

**Keywords:** antimicrobial resistance, *bla*_KPC-2_, *bla*_NDM-1_, *bla*_VIM-24_, carbapenemase, plasmids, *Providencia hangzhouensis*

## Abstract

*Providencia hangzhouensis* is a recently described species within the *Providencia* genus that is most closely related to *Providencia rettgeri* and is infrequently isolated from human infections. Its clinical relevance and epidemiology remain poorly understood. Here, we describe two *P. hangzhouensis* isolates recovered from urine specimens from two epidemiologically linked adult patients. Carbapenemase production in both isolates was determined using the modified carbapenem inactivation method. The presence of carbapenem resistance genes, *bla*_NDM-1_, *bla*_KPC-2_ and *bla*_VIM-24_, was identified by multiplex real-time PCR and whole-genome sequencing. Both isolates belong to the novel ST222 and carry all three carbapenem resistance genes on an IncC plasmid. Genetic environment analysis revealed complex integration of mobile genetic elements; notably, *bla*_NDM-1_ was localized within the variable region of an insertion sequence common region 1 element, *bla*_KPC-2_ was located within a putative Tn3-like transposon and *bla*_VIM-24_ was identified as a gene cassette within a second class 1 integron. Epidemiological data uncovered evidence of horizontal transfer of the IncC plasmid involving a *Citrobacter freundii* ST185 isolate. The convergence of multiple antimicrobial resistance genes, including those conferring resistance to last-line antimicrobials, underscores the potential threat posed by this emerging drug-resistant pathogen.

**Impact Statement**

*Providencia hangzhouensis* remains systematically underdiagnosed in clinical laboratories, largely owing to phenotypic and genotypic similarities to its closest phylogenetic relative, *Providencia rettgeri*. In this study, we recovered two carbapenemase-producing *P. hangzhouensis* isolates from two epidemiologically linked patients. Using both short- and long-read whole-genome sequencing, we uncovered a unique resistance architecture in both isolates, consisting of three carbapenemase genes (*bla*_NDM-1_, *bla*_KPC-2_ and *bla*_VIM-24_) convergent on a single predicted conjugative IncC plasmid. Notably, *bla*_NDM-1_ was embedded within an insertion sequence common region 1-associated variable region integrated adjacent to a class 1 integron. Such genetic configuration is rarely reported in the literature. Additionally, we uncovered evidence of horizontal transfer of the IncC plasmid involving a *Citrobacter freundii* isolate, highlighting its potential to facilitate wider dissemination. Comparative genomic analysis of global *P. hangzhouensis* reveals that 79.85% of strains carry at least one carbapenem resistance gene, positioning this species as a potential source of resistance determinants against last-line antimicrobials. Collectively, these findings underscore the vital role of genomic surveillance for the detection and monitoring of emerging pathogens.

## Data Summary

Long-read assemblies and sequencing reads from both Illumina and Oxford Nanopore Technologies for *Providencia hangzhouensis* isolates PH1 and PH and *Citrobacter freundii* CF1 have been deposited in the National Centre for Biotechnology Information under BioSamples SAMN45877809, SAMN45458561 and SAMN49005325, respectively.

## Introduction

The genus *Providencia* consists of ubiquitous, Gram-negative, rod-shaped bacteria that are occasionally reported in human infections. Bacteria of this genus have been isolated from many environments including soil, water, sewage, insects, nematodes, crustaceans, animals and humans [1, 2]. Among the species of *Providencia*, *Providencia rettgeri* and *Providencia stuartii* are the most common causes of human healthcare-associated infections, typically associated with urinary tract infections (UTIs) [1, 3]. *P. rettgeri* plays an increasingly prominent role, largely due to its rising prevalence in healthcare settings and its persistence on medical devices [1]. However, many isolates previously classified as *P. rettgeri* actually belong to the recently described *Providencia hangzhouensis*, which shares ~92% average nucleotide identity (ANI) with *P. rettgeri* [4]. While much is still being understood about the epidemiology of *P. hangzhouensis*, this species has been recovered from environmental, animal and human matrices. In the clinic, they are known to be associated with multiple infections such as UTIs, bacteraemia, liver dysfunction and acute kidney injury [4–6].

*P. hangzhouensis* isolates are genetically diverse, driven in part by extensive recent recombination [4]. Furthermore, the presence of an open pangenome within this species indicates a greater likelihood of discovering additional novel genes as more genomes are identified [4]. This genetic plasticity may also facilitate the acquisition and spread of antimicrobial resistance. A few available reports indicate that *P. hangzhouensis* is resistant to commonly used antibiotics, including last-line therapeutics such as carbapenems [5, 6]. The emergence of multidrug-resistant strains, particularly those resistant to carbapenems, complicates treatment options and often necessitates high-dose combination drug therapy [5]. The most frequently detected carbapenem resistance mechanisms in *P. hangzhouensis* are the imipenemase metallo-beta-lactamase, encoded by the *bla*_IMP_ gene, and the New Delhi metallo-beta-lactamase, encoded by *bla*_NDM_ [4]. Carbapenemase genes are often carried on transmissible mobile genetic elements, such as plasmids. Their presence on mobile genetic elements may significantly complicate infection prevention and control efforts owing to their potential for transfer between bacterial species and into antimicrobial-susceptible organisms. Hence, understanding the acquisition and spread of carbapenemases is critical for informing infection prevention and control strategies and curbing the escalating threat of drug-resistant bacterial infections, thereby protecting the effectiveness of life-saving antimicrobials.

In this study, we used phenotypic techniques to determine antimicrobial susceptibility and detect carbapenemase activity and applied both short- and long-read whole-genome sequencing (WGS) technologies to characterize *P. hangzhouensis* isolates from two epidemiologically linked patients. These isolates harboured three distinct carbapenemase genes located on highly similar IncC plasmids. In both plasmids, *bla*_NDM-1_ was localized within variable gene region of an insertion sequence common region 1 (ISCR1) adjacent to a class 1 integron, *bla*_VIM-24_ was identified as a gene cassette within a second class 1 integron and *bla*_KPC-2_ was located within a putative Tn3-like transposon.

## Methods

### Epidemiological background and MALDI-TOF identification of clinical isolates

Two *Providencia* isolates, designated PH1 and PH2, were recovered from Patient A and Patient B, respectively, 2 days apart and ~1 month after sharing a room at a skilled nursing facility in the autumn of 2024. Both patients were medically complex with histories of complex resistant UTIs and indwelling urinary devices prior to their overlapping admission. Patient B had a history of hospitalization outside the United States for UTI ~8 months prior to specimen collection and had received multiple rounds of antibiotics, including a 2-week course of intravenous ertapenem for urosepsis/pyelonephritis. At the time of specimen collection, Patient B was not considered symptomatic of UTI but was receiving a long course of oral antibiotics for *Clostridioides difficile*; Patient A was admitted to the hospital displaying pyuria, dysuria and suprapubic pain, but was afebrile. Both patients experienced poor clinical outcomes following diagnosis. Patient A entered hospice care, discontinued antibiotic therapy and expired approximately a month after specimen collection, whereas Patient B continued to require repeated hospitalizations and antibiotic treatment for complex UTIs and associated complications. A *Citrobacter freundii* urine isolate was recovered from Patient B in mid-2025.

The isolates were sent to the New York State Department of Health Wadsworth Center, which is the Northeast Regional Laboratory for the Antimicrobial Resistance Laboratory Network (AR Lab Network) funded by the Centers for Disease Control and Prevention (https://www.cdc.gov/antimicrobial-resistance-laboratory-networks/php/about/domestic.html), for further characterization as a funded activity of this network to provide surveillance of carbapenem-resistant bacteria. Both *Providencia* isolates were initially identified as *P. rettgeri* using MALDI-TOF MS.

### Antimicrobial susceptibility testing and detection of carbapenemase activity

Minimum inhibitory concentrations (MICs) were determined using two methods. The Sensititre MDRGN4F plate (Thermo Fisher Scientific, East Grinstead, UK) was used to determine susceptibility to amikacin, ceftazidime-avibactam, ceftolozane–tazobactam, imipenem, levofloxacin and meropenem. Gradient diffusion strips (Liofilchem, Waltham, USA) were used to determine the MICs of aztreonam–avibactam and meropenem–vaborbactam. MICs were interpreted according to the Clinical and Laboratory Standards Institute guidelines [7].

Phenotypic tests using the modified carbapenem inactivation method (mCIM) were used to detect carbapenemase production in the isolates. mCIM results were interpreted using zone diameter criteria: 6–15 mm (positive), 16–18 mm (indeterminate) and ≥19 mm (negative) [8]. The presence of specific carbapenemase genes (*bla*_KPC_, *bla*_NDM_, *bla*_IMP_, *bla*_OXA-48_-like and *bla*_VIM_) was determined using multiplex real-time PCR assays developed at the Wadsworth Center and the Centers for Disease Control and Prevention [9].

### WGS and data analysis

Genomic DNA was extracted from the isolates using a QIAcube HT, and sequencing was performed on an Illumina NextSeq as previously described [10]. An in-house developed bioinformatic pipeline was used to characterize both isolates [11]. Briefly, sequencing reads were trimmed, and adapter sequences were removed using Trimmomatic v0.39 [12]. *De novo* genome assembly was performed on the paired-end read datapaired-reads data using SPAdes v4.0.0 [13]. The quality of the genome assemblies was assessed using quantitative measurements in QUAST v5.3.0 [14], CheckM2 [15] and BUSCO v5.8.2 [16]. The genome assemblies of both isolates were of high quality, each showing 100% completeness and 0.51% contamination. Both genome assembly lengths were 4.73 Mb and comprised of 81 contigs for PH1 and 80 for PH2, with N50 values of 233016 and 291597, respectively.

The isolates were also sequenced using long-read Oxford Nanopore sequencing (Oxford Nanopore Technologies, Oxford, UK) to generate complete genomes and characterize mobile genetic elements. Genomic DNA was extracted from bacterial pellets using the DSP Virus kit on an EZ1 automated extractor (QIAGEN, Hilden, Germany) and quantified using a Qubit fluorometer (Thermo Fisher Scientific, Waltham, MA). Sequencing libraries were prepared using the rapid barcoding kit (SQK-RBK114-96) according to the manufacturer’s instructions with minor modifications: replicate libraries were prepared from diluted DNA, with each replicate barcoded independently. The samples were then pooled and sequenced on a FLO-MIN114 flow cell on a GridION sequencer running MinKNOW v24.06.14 using super-accurate basecalling with Dorado v7.4.13. The long reads were filtered to keep only high-quality reads using Filtlong v0.2.1 (https://github.com/rrwick/Filtlong) with a minimum read length of 1,000 bp. Filtering was set to retain either 700 megabase pairs (~100× genome coverage) or 95% of the reads, whichever was more stringent. Hybrid genome assemblies were generated using Unicycler v0.5.0 with the default settings [17].

### Confirmation of species identity and genome relatedness

Initial *in silico* bacterial species identification was performed using Kraken2 v2.1.3 with the Kraken2 Standard 16 GB database [18]. Our Kraken analysis determined both isolates to be *P. rettgeri*. To validate the species identity, FastANI v1.34 [19] and the *in silico* digital DNA–DNA hybridization (dDDH) analysis [20] were performed. FastANI was used to calculate the genome-wide ANI for each genome in this study against all known *Providencia* species in the NCBI Prokaryotic Genome Database (https://www.ncbi.nlm.nih.gov/datasets/genome/#!/prokaryotes/). The dDDH index, which estimates genome-to-genome distances between genome pairs, was then calculated against the top FastANI match. Species identification is often refined using pairwise ANI with a threshold of ≥96% and dDDH with a threshold of ≥70.0% [4, 20].

Genome relatedness was assessed using the current version of an in-house pipeline LegioCluster 3, which clusters isolates on the basis of their genetic distance from internally selected reference genomes and evaluates relatedness through mutation event analysis [21].

### *In silico* sequence typing of *P. hangzhouensis*

*In silico* multilocus sequence typing (MLST) analysis was performed on the genome assemblies using mlst v2.23.0 (https://github.com/tseemann/mlst). Both genomes having novel sequence combinations of the five housekeeping alleles – *fusA*, *gyrB*, *ileS*, *lepA* and *leuS* – were submitted to the PubMLST *Providencia* database (https://pubmlst.org/organisms/providencia-spp) for sequence type (ST) assignment [22]. The plasmid replicon genes were identified and characterized using MOB-suite v3.1.9 [23] and ABRicate v1.0.1 (https://github.com/tseemann/abricate) with the PlasmidFinder [24] database using default settings. The genome assemblies were screened for the presence of antimicrobial resistance determinants using the AMRFinderPlus v3.12.8 and its accompanying resistance gene database developed by the National Center for Biotechnology Information (NCBI) [25].

### Phylogenetic tree construction

To contextualize the two genomes from this study with other *P. hangzhouensis* isolates from other locations, reads and genomes labelled as ‘*Providencia*’ in the NCBI’s short reads archive and genome server, respectively, were retrieved (as of April 2025). The reads were assembled as previously described. To ensure that only *P. hangzhouensis* genome assemblies were included, we used FastANI v1.34, as described above, to screen the publicly available genome assemblies. To ensure that only high-quality genomes were included in this study, the publicly available genomes were screened using QUAST and CheckM2, as described earlier, to include only genomes with <500 contigs, completeness of >95% and contamination of <5% (Table S1, available in the online Supplementary Material, Fig. 1). Genomes were annotated using Prokka v1.14.6 [26]. The annotated genomes were used as input files to estimate the pangenome (i.e. the totality of genes of all the genome assemblies in the dataset) using Panaroo v1.5.1 [27]. SNPs were extracted from the core genome alignment using SNP-sites v2.5.1 [28]. The core SNP alignment was then used as input for generating a maximum likelihood phylogenetic tree using RAxML v8.2.13 with the GTR+Gamma model of nt substitution [29, 30]. The resulting phylogeny was visualized and annotated using FigTree v1.4.4 (http://tree.bio.ed.ac.uk/software/figtree/) and Interactive Tree of Life (iTOL) v6.8.1 [31]. The core genome alignment and phylogenetic tree were used as inputs for partitioning genomes into sequence clusters of genetically similar individuals with the Bayesian hierarchical clustering algorithm FastBAPS v1.0.8 [32]. FastBAPS implements a rapid agglomerative clustering of aligned sequences using a pairwise SNP distance matrix and then applies a Bayesian model selection to determine which clusters should be merged at each step of the hierarchical process [32].

**Fig. 1.**
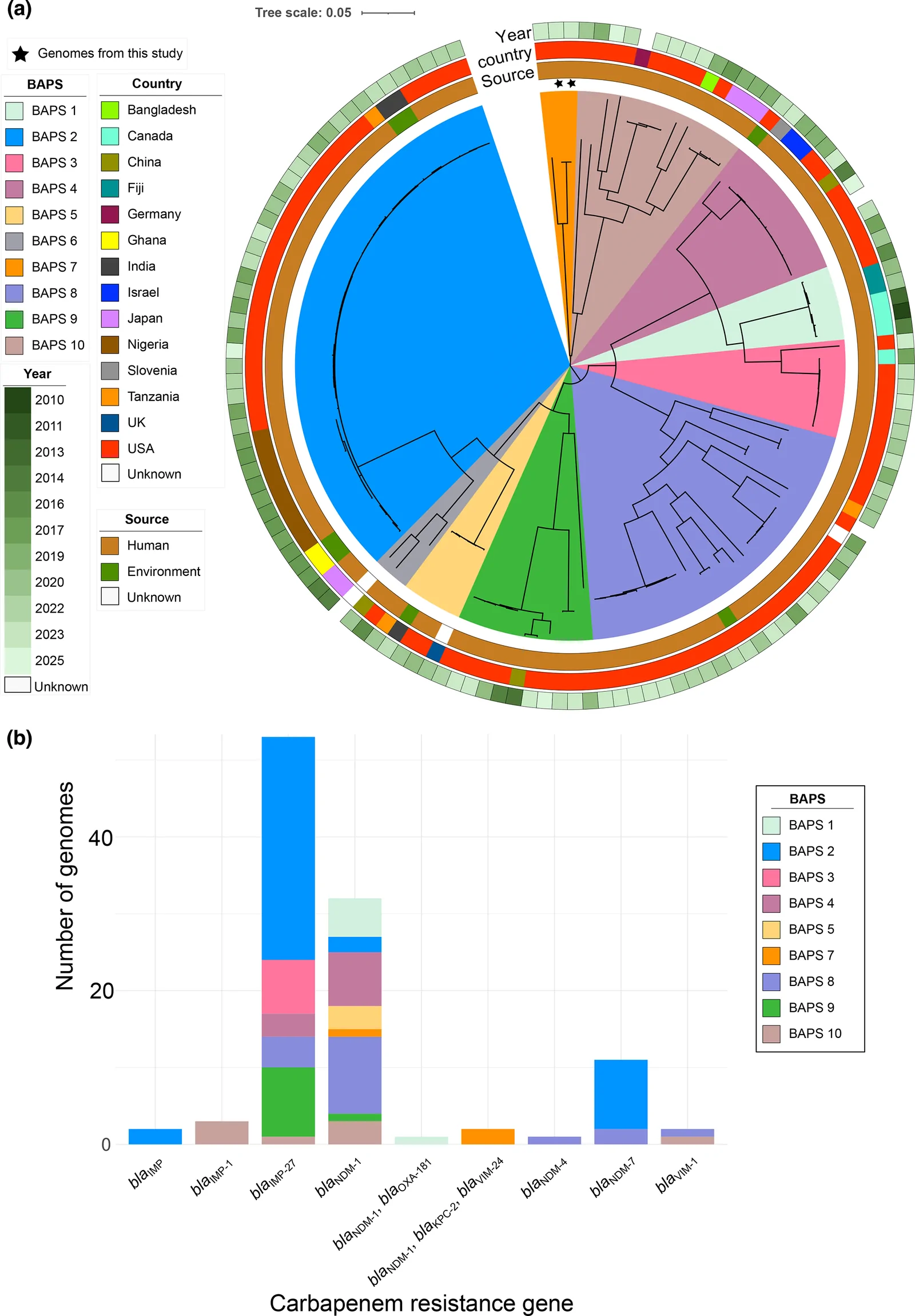
Global distribution of *P. hangzhouensis* and associated carbapenemase genes. (**a**) A maximum likelihood phylogenetic tree based on 87,758 SNPs extracted from a 3.39 Mb sequence alignment of 3,210 core genes. The tree is rooted at the midpoint. Tree scales indicate the number of nt substitutions per site. The tree clades are coloured by BAPS clusters. Black stars indicate the two genomes sequenced in this study. Coloured circular strips next to the tree show isolation source, country of isolation and year of isolation. (**b**) Bar chart showing the number of genomes carrying unique carbapenemase genes and gene combinations. Bars are coloured according to the BAPS clusters of the representative genomes.

### *In silico* plasmid genome sequence analysis

Minimap2 v2.30 [33] and SAMtools v1.21 [34] were used to map Oxford Nanopore reads for each isolate to the assembled IncC plasmid. The plasmid-derived reads were obtained and aligned to NCBI reference sequences of *bla*_KPC-2_ (NG_049253.1), *bla*_NDM-1_ (NG_049326.1) and *bla*_VIM-24_ (NG_050350.1). Mapping statistics were used to confirm the presence of each carbapenemase gene on the plasmid and assess its coverage and mean sequencing depth.

Hybrid genome assembly graphs were inspected for contiguity, completeness and circularity using Bandage [35]. MOB-suite v3.1.9 was used to predict plasmid mobility [23] from plasmid genome sequences. MOB-suites’s mobility prediction relies on the detection of relaxase, mate-pair formation and *oriT* sequences. Plasmid genomes are classified as conjugative if they contain both a relaxase and a mate-pair formation marker [23]. pyGenomeViz was used to visualize structural similarities between the plasmid genome assemblies (https://github.com/moshi4/pyGenomeViz). To identify plasmid genomes similar to those found in this study, all publicly available sequences in the PLSDB database were retrieved (as of April 2025) [36]. The publicly available plasmids were clustered using Mash v2.3 [37]. Mash distances between plasmids from this study and plasmids from the PLSDB were determined. A Mash distance threshold of 0.001 was used to cluster similar plasmid sequences [38, 39]. Proksee was used to visualize circular plasmid genomes by applying default parameters [40]. Within Proksee, mobileOD-db v1.1.3 was used to identify coding sequences associated with replication/recombination/repair, transfer, integration/excision and phage-related elements. CARD-RGI v1.3.0 was used to annotate antimicrobial resistance genes (https://card.mcmaster.ca/analyze/rgi).

## Results

### Genomic characterization and phylogenetic context of two *P. hangzhouensis* strains initially identified as *P. rettgeri*

Two isolates, PH1 and PH2, were recovered 2 days apart in 2024 from the urine samples of two adult patients. Both isolates were initially identified as *P. rettgeri* using both phenotypic (MALDI-TOF MS) and genotypic (Kraken taxonomic software). Since *P. rettgeri* is commonly misidentified [4], further genome sequence identity validation was carried out. Results revealed that when compared with the *P. rettgeri* type strain NCTC11801 (GCA_900455085.1), the strains PH1 and PH2 presented ANI values of 92.07 and 92.10%, respectively, and dDDH values of 9.39 and 9.40%, respectively. These values fall below the accepted threshold for determining isolate pairs of the same species (ANI ≥96%; dDDH >70%) [4, 19, 20]. Comparative ANI and dDDH analyses of all known *Providencia* type strains identified both PH1 and PH2 as *P. hangzhouensis*, with ANI and dDDH values of 98.7 and 95.5%, respectively. Both *P. hangzhouensis* isolates were genotypically similar to each other, differing by two SNPs. Both isolates had novel configurations of the five-gene MLST loci and were designated ST222 on the *Providencia* species database in the PubMLST database.

To place our *P. hangzhouensis* genomes in a broader context, we compared them to 132 publicly available *P. hangzhouensis* genomes. These publicly available genomes span across at least 14 countries, with the majority from the USA (*n*=92, 69.69%), and had isolation dates between 2010 and 2025 that ranged across human, animals and environmental sources (Table S1). The ANI between all genome pairs in the global dataset (*n*=134 including genomes from this study) ranged from 97.82to 99.9%. The genome sizes ranged from 4.3 to 5.1 Mb (median=4.71 Mb), with contamination levels between 0.12 and 2.62% (median: 0.47%) and 100% completeness across all assemblies (Fig. S1). Pangenome analysis identified a total of 13,062 genes, categorized into 3,210 core genes, 179 soft core genes, 1,748 shell genes and 7,925 cloud genes. A maximum likelihood phylogeny from 87,758 SNPs extracted from a 3.39 Mb sequence alignment of the core genes revealed a genetically diverse population (Fig. 1). The population consisted of 21 unique STs in 107 genomes (Simpson’s diversity index=0.826), whereas 27 genomes had no ST information. A Bayesian clustering algorithm implemented in FastBAPS [32] was used to delineate the genomes into genetically distinct phylogenetic sequence clusters. A total of 10 clusters were identified in the population (Simpson’s diversity index=0.813). Both genomes from this study belonged to cluster 7 (BAPS 7), together with another genome sequenced at the Wadsworth Center (SRR29580799) – an ST116 isolate carrying *bla*_NDM-1_ recovered in 2024 from a urine sample. The genomes from this study and SRR29580799 differed by 8823 pairwise core genome SNPs.

### Antimicrobial susceptibility and distribution of carbapenem resistance genes in *P. hangzhouensis*

Isolates PH1 and PH2 were tested for susceptibility to eight antimicrobials representing six drug classes. The isolates were resistant to amikacin (aminoglycosides), levofloxacin (fluoroquinolones), imipenem (carbapenems) and the cephalosporin/beta-lactamase inhibitor combinations ceftolozane–tazobactam and ceftazidime–avibactam. PH1 exhibited intermediate susceptibility to meropenem, whereas PH2 was resistant. Both isolates showed increased susceptibility to meropenem combined with the beta-lactamase inhibitor vaborbactam (Table S2). Both isolates were also susceptible to the monobactam/beta-lactamase inhibitor drug combination, aztreonam-avibactam.

Further testing demonstrated that both isolates were positive by the mCIM assay (7 mm), indicating carbapenemase production. Multiplex real-time PCR detected three unique carbapenem resistance genes (*bla*_NDM_, *bla*_KPC_ and *bla*_VIM_), which were further confirmed and variants determined as *bla*_NDM-1_, *bla*_KPC-2_ and *bla*_VIM-24_, by *in silico* analysis of the whole-genome assemblies. In total, 16 and 17 unique antimicrobial resistance genes were identified in isolates PH1 and PH2, respectively, spanning multiple antimicrobial drug classes. The antimicrobial resistance genes detected in both isolates, along with their corresponding drug classes/subclasses, include aminoglycoside [*aph(3′)-VIa*, *ant(2″)-Ia*, *aac(6′)-Ib-cr5*], beta-lactam (*bla*_TEM_, *bla*_OXA-1_), carbapenem (*bla*_KPC-2_, *bla*_VIM-24_ and *bla*_NDM-1_), bleomycin (*ble*), macrolide [*msr(E*)], macrolide/streptogramin [*mph(E*)], phenicol (*catB3*), rifamycin (*arr-3*), sulphonamide (*sul1*, *sul2*) and trimethoprim (*dfrA17*), whereas isolate P2 has an additional quinolone resistance gene, *qnrD1*, in its genome.

Carbapenemase genes were widely distributed in the global *P. hangzhouensis* dataset (Fig. 1b). A total of 79.85% (*n*=107/134) of the global strains possessed at least one carbapenem resistance gene (Table S1). The most widely distributed carbapenemase gene was *bla*_IMP_ (*n*=58/107, 54.20%), comprising *bla*_IMP-27_ (*n*=53/107, 49.53%), *bla*_IMP-1_ (*n*=3/107, 2.80%) and two uncharacterized *bla*_IMP_ variants. Forty-seven genomes carried *bla*_NDM_ (*bla*_NDM-1_=35, *bla*_NDM-4_=1 and *bla*_NDM-7_=11), whereas *bla*_VIM-1_ was detected in one genome. Excluding the two genomes from this study harbouring multiple carbapenemase genes, only one other genome in the global dataset carried more than one carbapenem resistance gene (*bla*_NDM-1_ and *bla*_OXA-181_) (Fig. 1b). The carbapenemase genes were sporadically distributed among the BAPS clusters with no single cluster dominating a carbapenemase gene family. However, no carbapenemase gene was detected in the BAPS6 cluster (Fig. 1b).

### Convergence of carbapenem and other antimicrobial resistance genes within mobile genetic elements on novel IncC plasmids with possible horizontal transfer to *C. freundii*

The two *P. hangzhouensis* isolates from this study underwent long-read Oxford Nanopore sequencing to determine the genetic context of the three carbapenem resistance genes and other antimicrobial resistance genes detected in their genomes. Mapping plasmid-derived long reads to the NCBI reference sequences of the carbapenemase genes confirmed their presence on the IncC plasmids of isolates PH1 and PH2. All references exhibited 100% coverage with a mean sequencing depth exceeding 65× (Table S3).

Isolates PH1 and PH2 harboured four and five circular plasmids, respectively, ranging in size from 2.6 to 194 kb (Table S4). In addition to the carbapenem resistance genes, 13 other antimicrobial resistance genes [*aph(3')-VIa*, *ant(2'')-Ia*, *aac(6')-Ib-cr5'*, *bla*_TEM_, *bla*_OXA-1_, *ble*, *msr(E*), *mph(E*), *catB3*, *arr-3*, *sul1*, *sul2* and *dfrA17*] were carried on IncC plasmids in both isolates. The IncC plasmids were 194,329 and 190,883 bp in length in PH1 and PH2, respectively. Both plasmids display high sequence homology with a pairwise ANI value of 99.99% (Fig. 2a). The IncC plasmids contained conjugal transfer genes, relaxase genes and an origin of transfer (*oriT*) and were predicted to be conjugative plasmids (Figs S2 and S3). The additional plasmid contained by isolate PH2 but not PH1 was a 5,310 bp Col3M replicon type harbouring a quinolone resistance gene, *qnrD1* (Fig. S4). We searched the PLSDB and the NCBI Nucleotide databases to identify plasmid sequences genetically similar to the IncC plasmids of the present study. This search yielded no positive hits. Additionally, the Mash distances between IncC plasmids from this study and all IncC replicon plasmids from the PLSDB (*n*=998 plasmid genomes) were above the threshold used to cluster similar plasmid sequences [38]. For instance, the most closely similar PLSDB plasmid (NZ_MZ465528.1) had a Mash distance of 0.009 and shared 697/1000 and 700/1,000 kmers with p1_PH1 and p1_PH2, respectively (Table S5), indicating that the IncC plasmids from this study likely contained a novel sequence configuration.

**Fig. 2.**
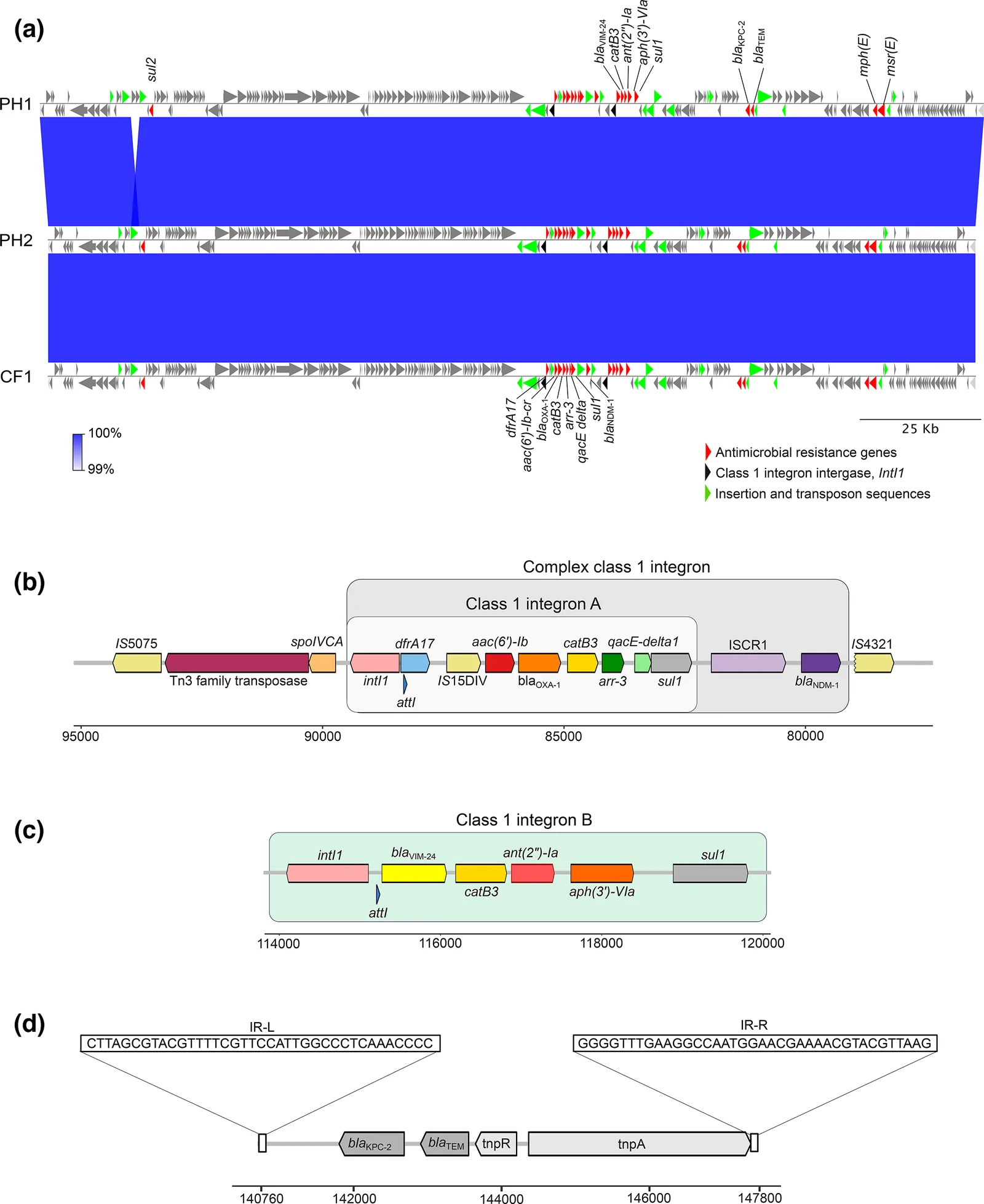
Mobile genetic elements and antimicrobial resistance genes associated with urinary *P. hangzhouensis*. (**a**) Plasmid sequence homology between isolates PH1, PH2 and CF1. The blue shaded regions represent regions of sequence similarity. Red coloured arrows represent antimicrobial resistance genes. The green coloured arrows represent insertion sequence elements, whereas the black coloured arrows represent class 1 integrases, *intI*. (**b**) Schematic sketch of genetic regions flanking *IS*5075 and *IS*4321 (truncated). The white shaded rectangle identifies genes identified in a class 1 integron (class 1 integron A), whereas the grey shaded rectangle identifies genes associated with a complex class 1 integron and its variable gene region. (**c**) Schematic showing genes identified in a second integron (class 1 integron B). (**d**) Schematic of putative Tn3-like transposon carrying *bla*_KPC-2_ and *bla*_TEM_ genes.

The genetic background of the carbapenemase genes and other antimicrobial resistance genes present in the IncC plasmid assemblies was closely examined. Two distinct class 1 integrons (class 1 integron A=~7 kb and class 1 integron B=~5.7 kb) which contained the conserved elements: the 5′ region with the integrase gene (*intI1*), the integrase recombination site (*attI*) and the 3′ conserved segment with *sul1*. While both integrons shared the *catB3* resistance cassette (Fig. 2b, c), they also harboured different unique cassettes. Class 1 integron A carried *aac(6′)-Ib-cr*, *bla*_OXA-1_ and *arr* (Fig. 2b), whereas class 1 integron B contained the carbapenemase gene, *bla*_VIM-24_, alongside *ant(2″)-Ia* and *aph(3′)-VIa* (Fig. 2c).

Class 1 integron A, together with *bla*_NDM-1_ and flanking transposable elements, formed a unique resistance gene cluster characteristic of a complex class 1 integron located within a defective transposon. The flanking elements, *IS*4321 and *IS*5075, were identified in opposite orientations; both belong to the IS1111 sub-family of the IS110 family transposase. While *IS*5075 was structurally intact with a 100% identity and inverted repeats (IRs) consistent with reference sequences from the ISfinder database [41], the *IS*4321 transposase was N-terminally truncated. Specifically, it lacked 60 aa that encode its *oriIS* (origin of replication) end, which likely renders the element incapable of transposition.

Upstream of *IS*4321, an IS91-like ISCR1 family transposase (1542 bp) was identified and connected to the 3′ end (sul1/qacE-delta1) of the class 1 integron A. The *bla*_NDM-1_ gene is localized within the ISCR1-associated variable region. This arrangement is consistent with the formation of a complex class 1 integron and represents a powerful gene-capturing system that facilitates the mobilization of diverse antimicrobial resistance genes [42–44].

The *bla*_KPC-2_ gene is located adjacent to *bla*_TEM_ gene within a Tn3-like transposon, comprising a 558 bp *tnpR* resolvase gene and a 3,009 bp *tnpA* transposase gene. Both 38 bp IRs characteristic of the Tn3-family transposase were identified [45]. The left IR (IR-L) is located at positions 14,7370–14,7407 downstream of *bla*_KPC-2_, whereas the right IR (IR-R) overlapped the *tnpA* stop codon by 5 bp. The IR-L and IR-R sequences shared 36 of 38 identical nt, and a perfect 5 bp target site duplication (TATGT) flanked the transposon boundaries. Together, these features are consistent with *bla*_KPC-2_ residing within an ~6.6 kb putative Tn3-like transposon.

We searched the entire ~6.6 kb putative transposon sequence against the NCBI Nucleotide database to identify publicly available genomes harbouring the same genetic architecture and to investigate its epidemiological distribution. The search yielded 11 hits with 100% coverage and ≥99.98% sequence identity (Fig. S5). These hits revealed that the putative transposon was present on plasmids (from *Escherichia coli* and *Klebsiella pneumoniae*) ranging from ~15 to 200 kb. These plasmids represented multiple replicon backbones, including IncN, IncN/IncR, ColRNAI and IncF (Table S6).

Lastly, we uncovered evidence suggestive of horizontal transfer of the IncC plasmid involving a *C. freundii* ST185 isolate, designated CF1, recovered from Patient B (Fig. 2a). Isolate CF1 harboured two plasmids: a 262,114 bp pKPC-CAV1321 replicon plasmid and a 190,833 bp IncC replicon plasmid (Table S4). The IncC plasmid in CF1 exhibits 100% nt sequence identity to the triple carbapenemase-encoding IncC plasmid identified in PH2 (Fig. 2a). Because CF1 was recovered from the same patient less than 1 year after PH2 was isolated, these findings suggest that horizontal transfer of the IncC plasmids occurred within the host, though the direction of that transfer cannot be confirmed.

## Discussion

The discovery of antibiotics represents a critical landmark in modern medicine. However, the rapid and indiscriminate bacterial acquisition of genetic determinants that undermine the efficacy of antimicrobial agents has become a severe and global public health problem. Mobile genetic elements play pivotal roles in the acquisition and dissemination of antimicrobial resistance genes, including those conferring resistance to last-resort drugs, such as carbapenems. In this study, we report the phenotypic and genotypic characterization of *P. hangzhouensis* isolates recovered from urine samples of hospital patients and harbouring multiple carbapenem resistance genes. To the best of our knowledge, this is the first report on the accumulation of three distinct carbapenemase genes in *P. hangzhouensis*.

The two genomes analysed in this study were initially identified as *P. rettgeri* via phenotypic and genotypic methods. This misclassification was not unexpected as *P. hangzhouensis* which shares the closest ANI with *P*. rettgeri was only recently characterized and was not available in databases used for identification at the time these isolates were first characterized [4]. For instance, only 15.47% of sequences deposited in the public databases were reported to be actually *P. rettgeri*, whereas 29.46% of the identified *P. rettgeri* were actually *P. hangzhouensis* [4]. This example, like others (e.g. [46, 47]), reflects an inherent issue when renaming clinically relevant organisms; misclassification of bacterial species can obscure our understanding of their clinical and epidemiological relevance. In addition, accurate bacterial taxonomic classification plays important roles in patient care, as it can influence the interpretation of antimicrobial susceptibility tests, guide appropriate therapy and aid in prognosis prediction. Whole-genome-based ANI analysis remains a robust and reliable method for accurately delineating the species boundaries among genetically similar strains [48].

Comparative genomics with publicly available *P. hangzhouensis* genomes revealed a genetically diverse population with extensive dissemination of carbapenemase genes within the species. These carbapenem-hydrolysing enzymes may compromise the efficacy of carbapenems, which are often reserved as last-resort treatments. Interestingly, carbapenemase genes are frequently carried on mobile genetic elements [49]. The widespread distribution of these genes in this species underscores the potential for *P. hangzhouensis* to contribute clinically relevant antimicrobial resistance genes, thereby highlighting its emerging public health importance. Despite this, *P. hangzhouensis* remains a relatively understudied species, underscoring the need for its inclusion in routine clinical genomic surveillance programmes.

The mCIM and antimicrobial susceptibility results for both *P. hangzhouensis* isolates were consistent with a multi-drug-resistant, carbapenemase-producing phenotype exhibiting resistance to multiple frontline antimicrobial classes while remaining susceptible to newer beta-lactam/beta-lactamase inhibitor combinations. Restoration of meropenem activity in the presence of vaborbactam, together with susceptibility to aztreonam-avibactam, suggests that these agents may represent viable therapeutic options. Both combinations have demonstrated high efficacy against carbapenem-resistant *Enterobacterales* causing complicated UTIs [50, 51]. Nevertheless, this resistance profile does highlight the clinical challenge posed by emerging carbapenemase-producing *P. hangzhouensis* and underscores the importance of comprehensive phenotypic characterization alongside genomic analysis to guide antimicrobial therapy.

Investigation of the genotypic basis of carbapenem resistance revealed that our isolates harboured three distinct carbapenemase genes, *bla*_NDM-1_, *bla*_KPC-2_ and *bla*_VIM-24_ which were embedded within IncC plasmids. Strains harbouring multiple carbapenemase genes have been discovered in many parts of the world [52, 53]. Specifically, the co-localization of KPC, NDM and VIM genes, as observed in our study, has been reported in *K. pneumoniae* [52]. These genes were identified using PCR, and hence, their genetic background could not be compared to ours. Plasmids carrying multiple carbapenemase genes are described to confer evolutionary advantage by enhancing the antibiotic resistance profile of their host bacteria and have been associated with poorer clinical outcomes [53]. Furthermore, IncC replicon plasmids (formerly known as IncA/C) are broad-host range, conjugative and are considered plasmids of public health concern as they tend to carry multiple genes that confer antimicrobial resistance [54, 55]. Our study was limited in our inability to perform laboratory-based mating experiments to experimentally validate the transferability and stability of the IncC plasmids; however, our genomic and epidemiological data provides compelling evidence of potential *in vivo* horizontal plasmid transfer involving a *C. freundii* ST185 isolate. The recovery of identical IncC plasmids from different bacterial species within the same patient within a 1-year period underscores likely mobility of this plasmid and its potential role in the proliferation of antimicrobial resistance. Overall, multi-drug resistance and conjugative plasmids, particularly those that confer resistance to carbapenems, represent a threat to global health and could accelerate the evolution and dissemination of antibiotic resistance in clinically relevant pathogens.

We identified an ISCR1 element in the resistance gene cluster of IncC plasmids in this study. ISCR elements represent a highly efficient gene-capturing system, first characterized by Toleman *et al*. [42]. As IS91-like elements, they utilize rolling circle transposition. This mechanism allows for the mobilization of large genetic segments located immediately adjacent to their 3′ end, effectively acting as a powerful engine for horizontal gene transfer [43]. In the present study, *bla*_NDM-1_ was identified within the variable region of ISCR1. The insertion of genes into the ISCR-variable region is described to occur via a phenomenon known as ‘unchecked transposition’, frequently as a result of misreading the termination sequence, *terIS* [42]. While ISCR1 has been widely linked to a variety of antimicrobial resistance genes such as class A, B and C beta-lactamases, aminoglycosides and quinolones, its co-location with *bla*_NDM_ remains relatively scarce in the literature [42, 56].

ISCRs are particularly associated with the formation of complex class 1 integrons [42, 57]. By providing an additional mechanism for gene acquisition beyond the standard integrase-mediated cassette system, these complex class 1 integrons facilitate the rapid assembly of multi-drug resistance profiles in clinical settings [44, 58, 59]. Downstream of this complex integron, we observed a truncated *IS*4321. Repeated mobilization of ISCR elements is often associated with whole or truncated sequences located adjacent to the *terIS* region of the element [43]. These truncated genes are considered hallmarks of ISCR-mediated transpositions from repeated *terIS* activity [43]. This may explain the presence of a truncated *IS*4321 adjacent to ISCR1 in our study.

In our global *P. hangzhouensis* analysis, only isolates from this study contained a *bla*_KPC-2_ gene, suggesting that this carbapenem resistance gene is relatively uncommon in this species. Further characterization of the genomic context of the *bla*_KPC-2_ gene revealed that it resides within a ~6.6 kb putative Tn3-like transposon. Tn3 family transposons are widely distributed and have been implicated in the dissemination of antimicrobial resistance determinants [60, 61]. Analysis of the genomic similarity and epidemiological distribution of this element revealed that the putative *bla*_KPC-2_-associated Tn3-like element was conserved across diverse plasmid backbones, including IncN, IncN/R, ColRNAI and IncF, but not IncC, and was identified in clinically important bacterial species. These findings suggest that the putative transposon may facilitate the dissemination of *bla*_KPC-2_ independently of the surrounding plasmid backbone, potentially broadening opportunities for its persistence and horizontal spread among bacterial hosts.

In summary, this study highlights the crucial role of integrated WGS for uncovering the genetic mechanisms of antimicrobial resistance in clinical and epidemiological surveillance. Through the application of long- and short-read sequencing, we revealed the unique arrangement of carbapenemase genes and other antimicrobial resistance genes within mobile genetic elements in IncC plasmids harboured by *P. hangzhouensis* and *C. freundii*.

## Supplementary material

10.1099/mgen.0.001876 Supplementary Material 1.

10.1099/mgen.0.001876 Supplementary Material 2.

## Abbreviations

ANI: average nucleotide identity
dDDH: digital DNA–DNA hybridization
IR-L: left inverted repeat
IR-R: right inverted repeat
ISCR1: insertion sequence common region 1
mCIM: modified carbapenem inactivation method
MICs: minimum inhibitory concentrations
MLST: multilocus sequence typing
NCBI: National Center for Biotechnology Information
ST: sequence type
UTIs: urinary tract infections
WGS: whole-genome sequencing
