# Supplementary material for: Genomic characterization of IncC plasmid-mediated triple carbapenemase-producing Providencia hangzhouensis ST222
Source: Microb Genom. 2026 Nov 10;12(11):001876. doi: 10.1099/mgen.0.001876 (PMC13750824; doi:10.1099/mgen.0.001876)
Supplement: Supplementary file 1 [file mgen-12-01876-s001.pdf]

Genomic Characterization of IncC Plasmid-Mediated Triple Carbapenemase-Producing *Providencia hangzhouensis* ST222

Odion. O. Ikhimiukor[1], Catharine Prussing[1], Kailee Cummings [1], Ashley L. Marcinkiewicz [1], Spencer Y. Kennedy[1], Jenna Cebelius[2], Elizabeth J. Nazarian[1], Kimberlee A. Musser[1]

[1]Wadsworth Center, New York State Department of Health, Albany, NY, United States.

[2]Vermont Department of Health, Waterbury, VT, United States

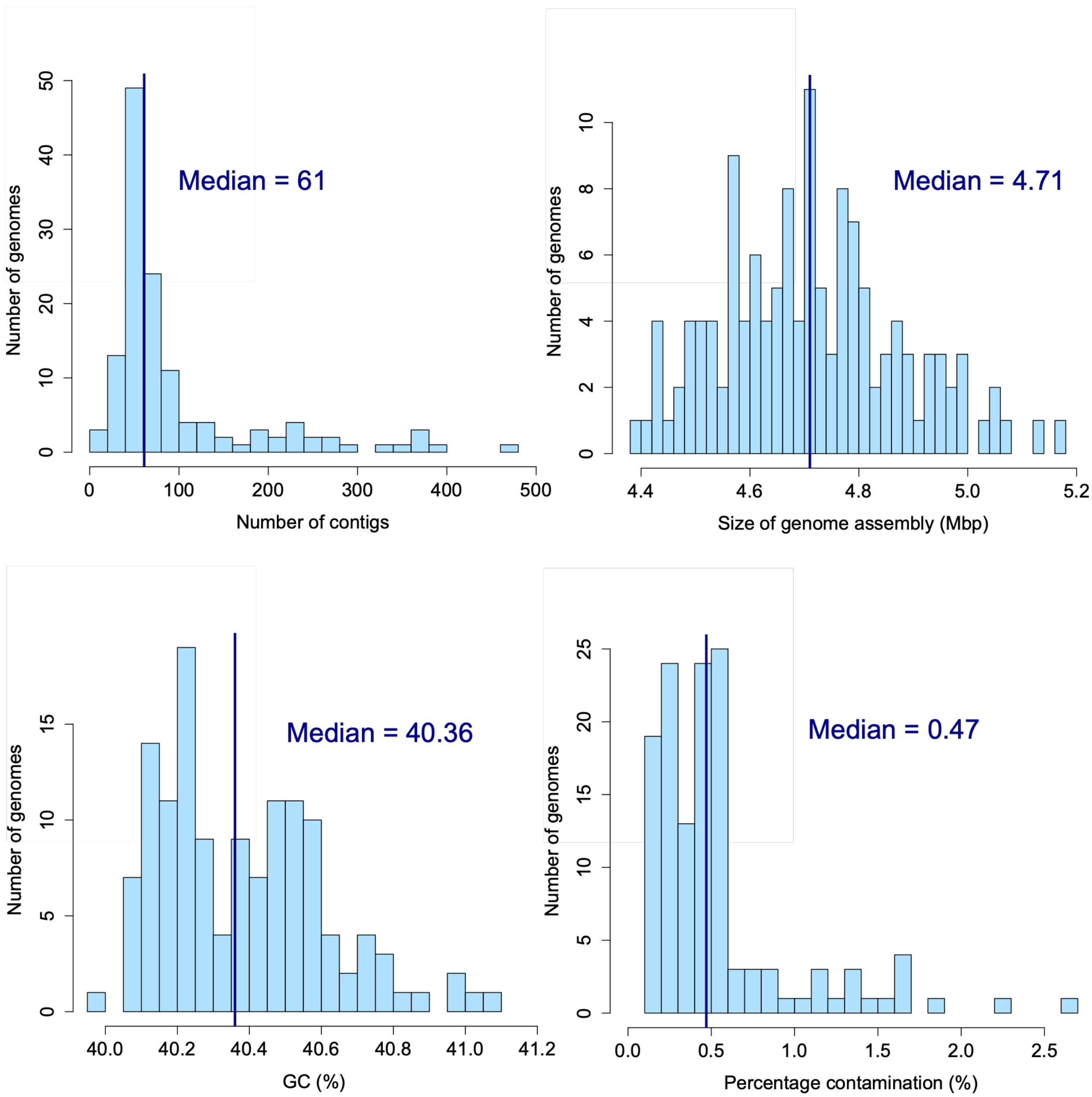

Supplementary Figure S1: Genome quality metrics of 134 *P. hangzhouensis* genomes (including the two isolates from this study) from public databases. (A) number of contigs, (B) size of genome assembly (C) G+C content, (D) Percentage contamination.

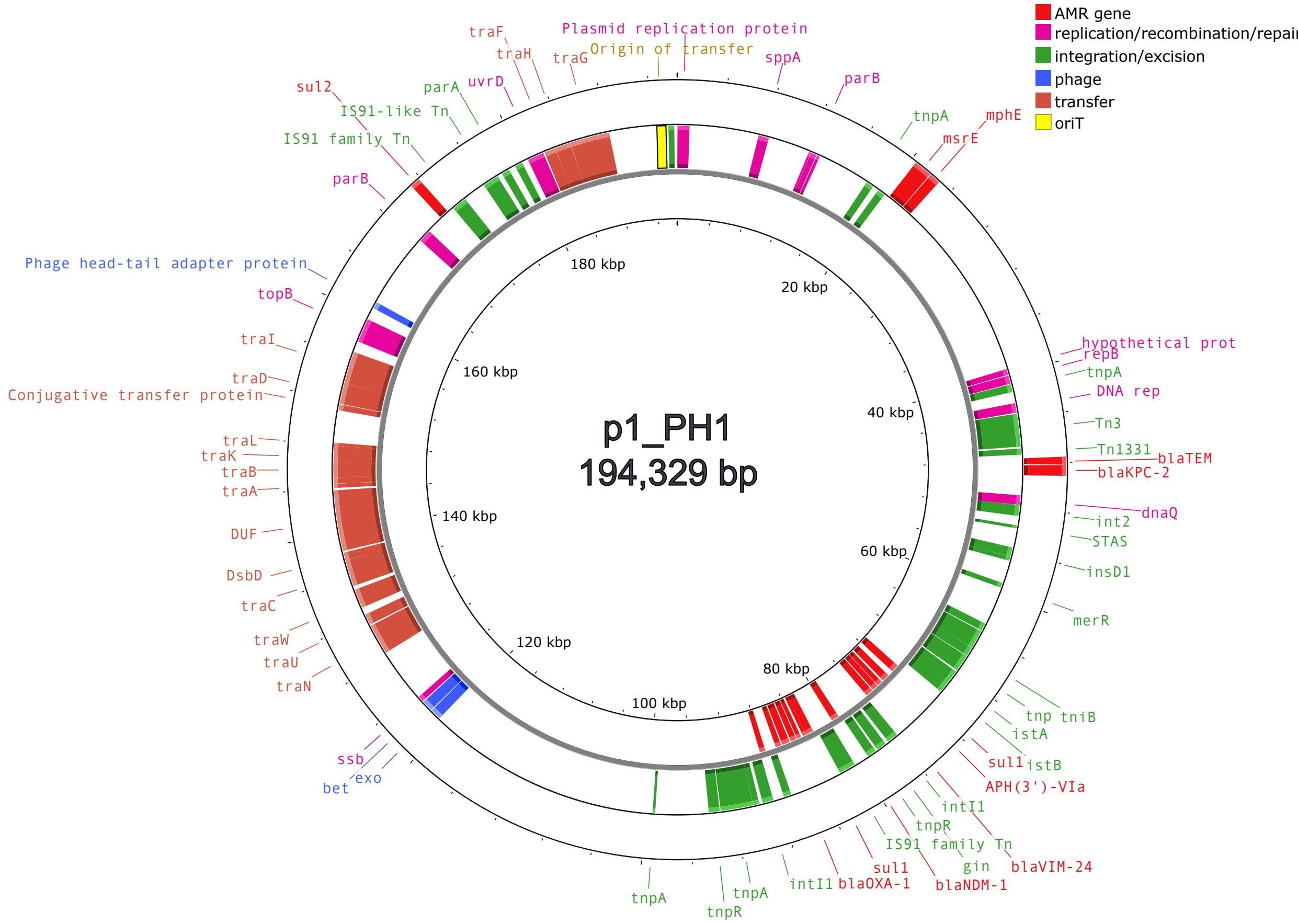

Supplementary Figure S2: Circular topology of plasmid genome assembly, p1_PH1, bearing *bla*NDM-1, *bla*KPC-2 and *bla*VIM-24 derived from *P. hangzhouensis* PH1 isolate sequenced in this study. The plasmid map shows antimicrobial resistance genes (labeled in red) and other genes involved in replication/recombination/repair, integration/excision, transfer, phage elements and *oriT* in plasmids.

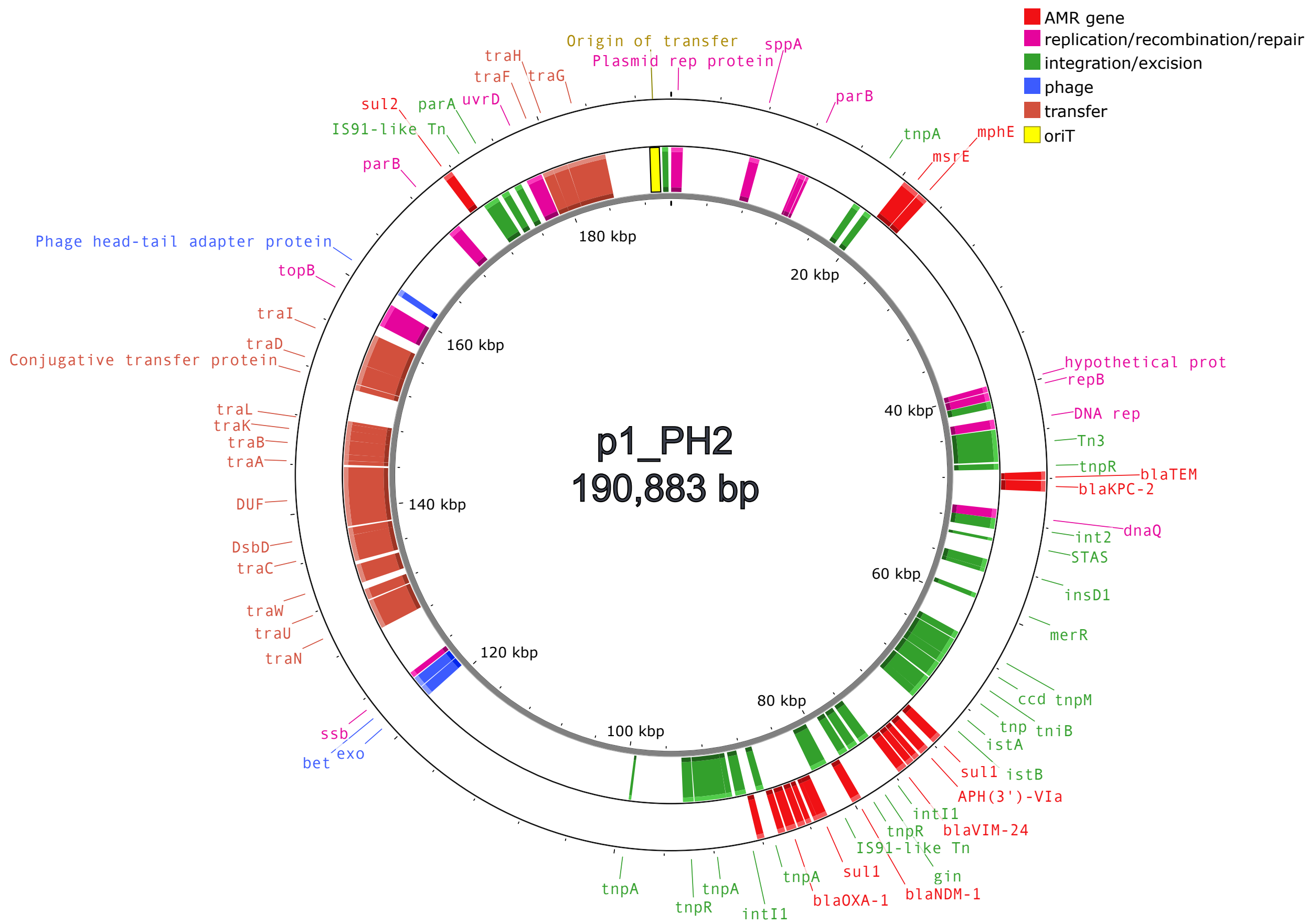

Supplementary Figure S3: Circular topology of plasmid genome assembly, p1_PH2, bearing *bla*$_{NDM-1}$, *bla*$_{KPC-2}$ and *bla*$_{VIM-24}$ derived from *P. hangzhouensis* PH2 isolate sequenced in this study. The plasmid map shows the antimicrobial resistance genes (labeled in red) and other genes involved in replication/recombination/repair, integration/excision, transfer, phage elements and *oriT* in plasmids.

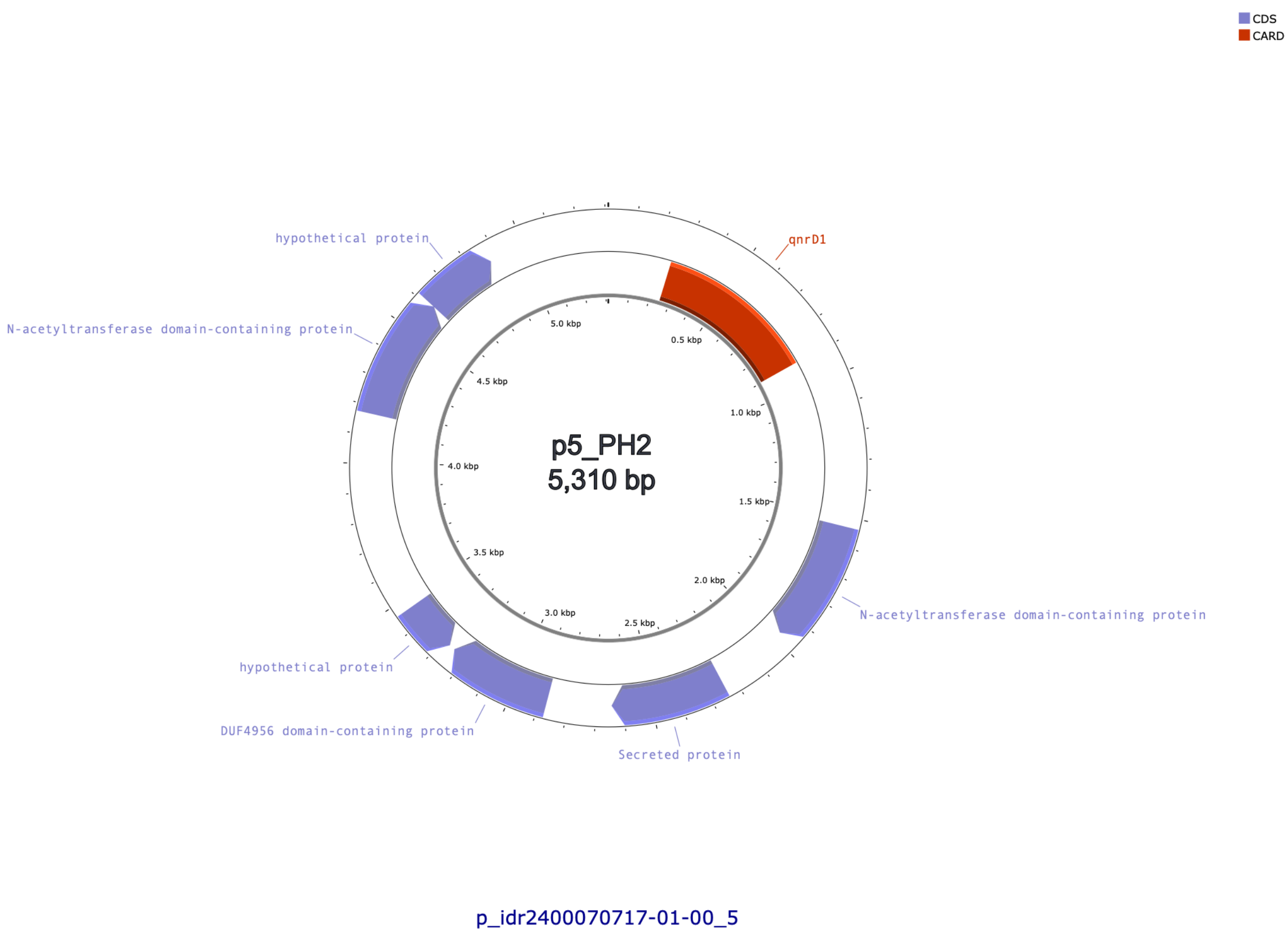

Supplementary Figure S4: Circular topology of plasmid genome assembly, p5_PH2, bearing *qnrD1* derived from *P. hangzhouensis* PH2 isolate sequenced in this study.

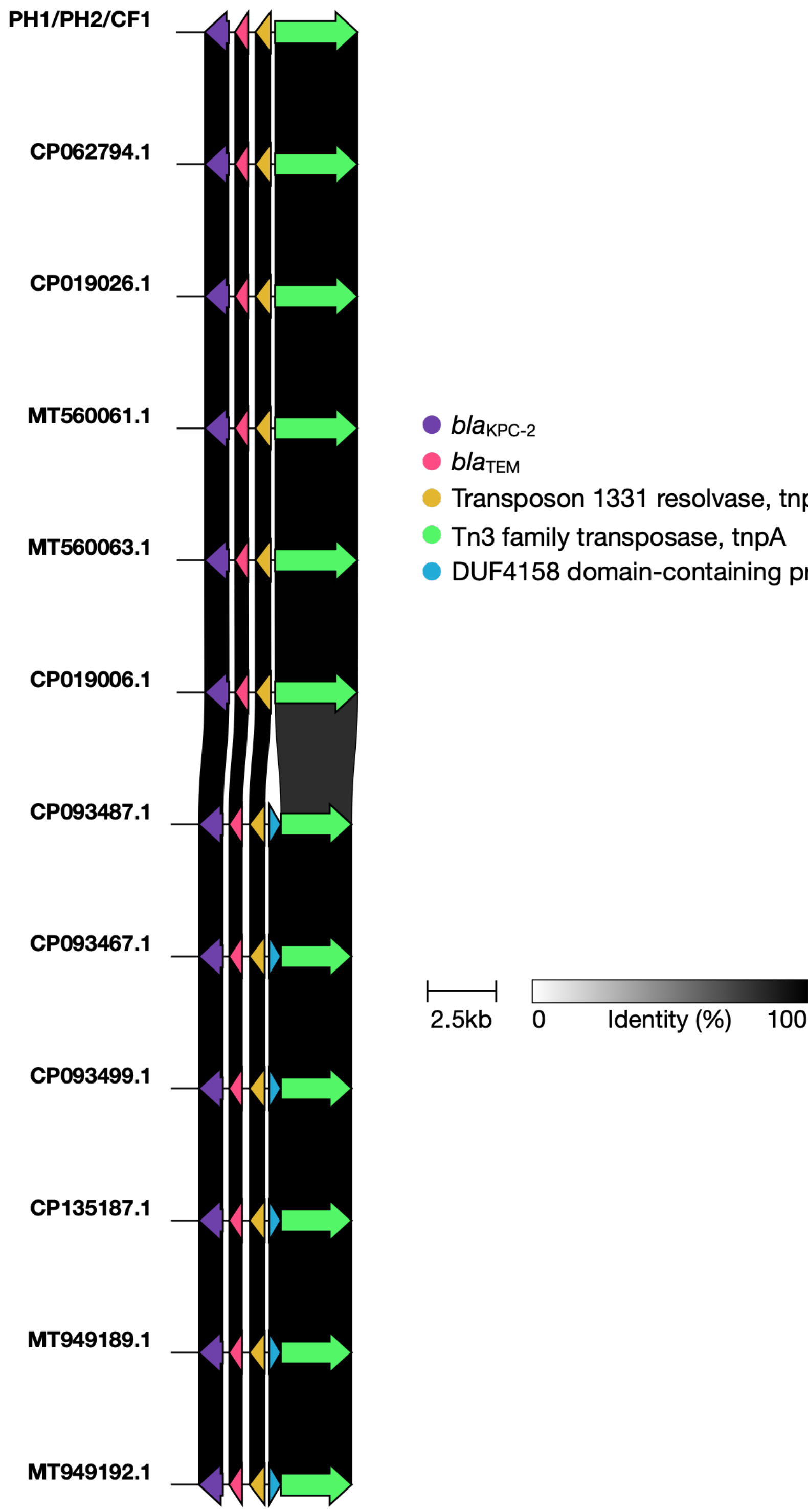

Supplementary Figure S5: Comparison of the genetic neighborhood of putative Tn3-like transposon in plasmids from this study and those from publicly available genomes.
